# Preschoolers Represent Abstract Relations Predicated on Kind Membership

**DOI:** 10.1162/opmi_a_00179

**Published:** 2025-01-04

**Authors:** Rebecca Zhu

**Affiliations:** Department of Psychology, Stanford University, Stanford, CA, USA

**Keywords:** relational reasoning, abstract relations, relational match to sample, kind representations, noun labels

## Abstract

Recent work demonstrates that U.S. preschoolers can represent the abstract relational concepts *same* and *different* when these abstract relational concepts are predicated upon perceptual dimensions (e.g., size, shape, color). The current research investigates whether preschoolers (*n* = 192; predominantly White, upper middle class, U.S. convenience sample) can also represent the abstract relational concepts *same* and *different* when these abstract relational concepts are predicated upon abstract dimensions (e.g., kind membership). Experiment 1 shows that, at baseline, 4-year-olds fail at a relational match-to-sample (rMTS) task with familiar kinds. However, Experiment 2 shows that 4- and 5-year-olds, but not 3-year-olds, succeed at a rMTS task with familiar kinds when provided with training involving noun labels. Experiment 3 shows that 4- and 5-year-olds also succeed at a rMTS task with novel kinds when provided with training involving noun labels but not adjective labels, suggesting that noun labels but not adjective labels cue children’s attention towards kind membership. Moreover, participants frequently provided explanations appealing to sameness and difference when justifying their responses. Taken together, these results suggest that, with training, preschoolers are capable of representing abstract relations predicated on abstract, as well as perceptual, dimensions.

## INTRODUCTION

Relational reasoning—the ability to go beyond perceptual similarity to recognize abstract relations between objects—is a key component of human cognition (Gentner, 2010). For example, relational reasoning facilitates problem solving in human adults (Gick & Holyoak, 1980; Novick & Holyoak, 1991) and plays a critical role in both generating and understanding some kinds of creative language, such as metaphors (Bowdle & Gentner, 2005; Gentner, 1988; Holyoak & Stamenković, 2018; Roberts & Kreuz, 1994; Wolff & Gentner, 2011; Zhu, Goddu, Zhu, et al., 2024; Zhu & Gopnik, 2023). Moreover, relational reasoning can be a powerful source of conceptual change in both everyday thinking (Lombrozo, 2019; Xu, 2019) as well as scientific theories (Carey, 2009; Nersessian, 2015). For example, historians of science have noted the importance of the abstract relational comparisons—specifically between “atoms” and “solar systems”—in the discovery of the structure of atoms (Kuhn, 1993). Similarly, relational reasoning is one kind of cognitive mechanisms for “learning by thinking,” which may be critical for the acquisition of new knowledge (Lombrozo, 2019). Specifically, mechanisms for learning by thinking may help both children and adults expand their existing conceptual repertoire, by going beyond existing data to generate new ideas (Xu, 2019). Overall, it is evident that relational reasoning is a powerful tool for human cognition for adults, and possibly also for children.

Recent work in developmental psychology suggests that relational reasoning is an early-emerging ability, already present in preschoolers (Brockbank et al., 2023; Carstensen et al., 2019; Christie & Gentner, 2014; Goddu et al., 2020; Hochmann et al., 2017; Holyoak et al., 1984; Kroupin & Carey, 2022b), toddlers (Walker et al., 2016; Walker & Gopnik, 2017), and even infants (Anderson et al., 2018; Hochmann, 2022; Hochmann et al., 2016). Indeed, infants and preschoolers can apply the abstract relations of sameness and difference not only along a single perceptual dimension, such as size or color (Goddu et al., 2020; Hochmann et al., 2018), but also across multiple perceptual dimensions simultaneously (Carstensen et al., 2019; Christie & Gentner, 2014; Hochmann et al., 2017; Kotovsky & Gentner, 1996). In other words, a wealth of literature shows that children can apply abstract relations to items sharing the same *perceptual identity*—that is, two items that are the same on all encoded perceptual dimensions (e.g., shape and color). For example, a classic paradigm investigating children’s relational reasoning capacities is the relational match-to-sample (rMTS) task (Premack, 1983). In a classic rMTS, participants are presented with a sample pair, and must match one of two choice pairs to the sample pair. Each pair contains two objects that have either the same or different perceptual identities. The correct choice pair is the one in which the relation between the objects matches the relation in the sample pair. For example, a same choice pair might consist of two blue squares (i.e., AA), whereas a different choice pair might consist of a green triangle and an orange circle (i.e., BC). If the sample pair consisted of two yellow stars (i.e., XX), then the correct answer would be the same choice pair consisting of two blue squares (i.e., AA). In contrast, if the sample pair consisted of a brown rectangle and a purple hexagon (i.e., YZ), then the correct answer would be the different choice pair consisting of a green triangle and an orange circle (i.e., BC). Hochmann et al. (2017) demonstrated that 5-year-olds, and some 4-year-olds, succeed at the classic rMTS predicated upon sameness and difference of perceptual identity. Moreover, Kroupin and Carey (2022b) demonstrated that, when given facilitatory training to change their inductive biases, 4-year-olds also succeed at a classic rMTS predicated upon sameness and difference of perceptual identity.

Indeed, the vast majority of developmental psychology research on relational reasoning examined infants’ and children’s capacity to represent sameness and difference predicated upon perceptual dimensions. In particular, researchers have explored children’s developing representations of abstract relations predicated upon the dimension of perceptual identity, across a wide variety of age ranges and paradigms. For example, researchers have shown that infants can habituate to either sameness or difference of perceptual identity, and dishabituate when later presented with the opposite relation (Anderson et al., 2018). Similarly, toddlers can learn to select pairs of the same perceptual identity, or pairs with different perceptual identities, to make a blicket detector light up (Walker et al., 2016; Walker & Gopnik, 2017). Some research demonstrates that preschoolers can also represent abstract relations predicated on the dimensions of color, size, and small numbers (i.e., the numbers 1 through 5; Goddu et al., 2020; Kroupin & Carey, 2022b), though children might succeed at the experimental paradigms involving the abstract dimension of number through their sensitivity to perceptual dimensions such as area (Odic et al., 2013). Overall, there is overwhelming evidence showing that young children can represent sameness and difference along perceptual dimensions.

While all relational concepts (e.g., the concepts *same* and *different*) are abstract, the features that enter into these relations can be either perceptual (e.g., same size, color, shape; Cavanaugh et al., 2016; Skelton et al., 2022; Smith & Kemler, 1977) or abstract (e.g., same structure, causal role, kind membership; Carey, 2009; Haward et al., 2018; Sobel et al., 2007). Critically, while abstract dimensions can sometimes correlate with perceptual dimensions, abstract dimensions can also sometimes diverge from perceptual dimensions. For example, members belonging to the same category (e.g., an apple and an orange) are typically more perceptually similar than members belonging to different categories (e.g., an apple and a cup). However, members belonging to the same category (e.g., apple and a banana) can also be more perceptually dissimilar than members belonging to different categories (e.g., an apple and a balloon) (Gentner & Namy, 1999). Importantly, human adults can represent sameness and difference predicated on both perceptual and abstract dimensions. Indeed, human adults can represent abstract relations predicated upon an *infinite* number of dimensions. Two concepts can be the same or different along an infinite number of dimensions that are not restricted to specific modalities or cognitive domains (e.g., two images, smells, sounds, animals, or ideas can be the same as—or different from—each other). Moreover, the capacity to represent abstract information may be particularly valuable (i.e., the so-called “blessing of abstraction”; Gershman, 2017; Goodman et al., 2011). In some instances, learning information at higher levels of abstraction may encourage further learning at lower levels. For example, learning about animals in general may license additional inferences about specific kinds of animals, like sloths or aardvarks (Ullman & Tenenbaum, 2020). Indeed, representing sameness and difference along abstract dimensions might be more useful for further thinking, reasoning, and learning than representing sameness and difference along perceptual dimensions, at least in some contexts (Zhu, Goddu, & Gopnik, 2024).

Given previous research demonstrating that preschoolers can represent the abstract relations of sameness and difference predicated upon *perceptual* dimensions, the current research investigates whether preschoolers can also represent the abstract relations of sameness and difference predicated upon an *abstract* dimension, namely kind membership. Previous research shows that infants and young children already possess a sophisticated, abstract understanding of kind membership (Dewar & Xu, 2007, 2009; Gelman, 2003). Preschoolers understand that members of the same kind share internal, causal similarities even in the presence of superficial, perceptual dissimilarities (Booth, 2014; Gelman & Markman, 1986; Sobel et al., 2007). Similarly, by 4 years of age, preschoolers differentiate between conceptually relevant properties of kinds (e.g., functional features of artifacts, like watches telling time) from arbitrary but frequent properties of kinds (e.g., perceptual features of artifacts, like watches having round faces) (Haward et al., 2018). However, it is unclear whether preschoolers are able to reason about the sameness or difference of abstract features, such as kind membership. Indeed, previous research suggests that young children sometimes struggle to represent sameness predicated upon more abstract features (i.e., same function) relative to sameness predicated upon more perceptual features (i.e., same shape) (Zhu, Goddu, & Gopnik, 2024). Young children also sometimes struggle to understand metaphors based on abstract underlying similarities (Silberstein et al., 1982; Winner et al., 1976; though see also Özçalışkan, 2007; Zhu & Gopnik, 2023). Overall, preschoolers possess a sophisticated, abstract understanding of kind membership that extends beyond perceptual properties; however, more work is necessary to investigate whether preschoolers can reason about sameness and difference along abstract dimensions such as kind membership.

Critically, language may play a privileged role in helping children attend to both abstract relations (Christie & Gentner, 2014) and kind membership information (Dewar & Xu, 2007, 2009). For example, Christie and Gentner (2014) found that providing preschoolers with language training, specifically training with the words “same” and “different”, facilitated their performance on a classic rMTS task predicated upon perceptual identity. Separately, count noun labels highlight kind membership from infancy onwards (Dewar & Xu, 2007, 2009; Waxman & Markow, 1995). Consequently, language may also facilitate preschoolers’ performance on the present task, a rMTS predicated upon same and different kinds. In particular, count noun labels might highlight a relevant dimension in the present task, specifically kind membership. However, language may also facilitate preschoolers’ performance on the present task in other ways: for example, any kind of simple verbal repetition (e.g., hearing the same word repeated while looking at pictures of two of the same animals; or hearing two different words while looking at pictures of two different animals) may serve to highlight the sameness or difference relations between two items. Thus, the present series of studies will investigate if, and how, language might facilitate preschoolers’ performance on a rMTS predicated upon same and different kinds.

Moreover, previous work has shown that preschoolers can use language to explicitly justify their responses on relational reasoning tasks, and that preschoolers’ justifications often align with their overall task performance (Esmer et al., 2023; Hochmann et al., 2017; Zhu & Gopnik, 2023). Hochmann et al. (2017) found that the majority of preschoolers who succeeded on a classic rMTS task predicated upon perceptual identity explained their responses by appealing to the words “same” and “different”. In contrast, preschoolers who failed the same task frequently provided irrelevant explanations, for example about the features of individual objects. Similarly, Esmer et al. (2023) found that preschoolers’ spontaneous production of words describing the features of individual objects (e.g., color, shape) during a rMTS task predicated upon perceptual identity correlated with worse performance on the rMTS task. Consequently, we will also ask preschoolers to justify their responses, to generate additional converging evidence that preschoolers are leveraging their relational reasoning capacities to solve the present rMTS task.

Thus, in the current series of experiments, we present preschoolers with a rMTS predicated upon same and different kinds (e.g., matching a sample pair of two cats to a choice pair of two frogs, over another choice pair of a dog and a cow). Experiment 1 shows that 4-year-olds fail at a rMTS predicated upon same and different familiar kinds. However, Experiment 2 shows that 4- and 5-year-olds, but not 3-year-olds, succeed at the same rMTS predicated upon same and different familiar kinds when provided with training involving noun labels. Moreover, Experiment 3 shows that 4- and 5-year-olds also succeed at a rMTS task predicated upon same and different novel kinds when provided with training involving noun labels, but not adjective labels. Overall, these results suggest that when provided with noun label training, preschoolers, from four years of age onward, are capable of representing abstract relations predicated on abstract, as well as perceptual, dimensions. Moreover, preschoolers frequently appeal to the words “same” and “different” when justifying their responses. Consequently, children’s capacity to potentially represent abstract relations predicated across wide variety of abstract and perceptual dimensions might be a powerful tool for thinking, reasoning, and learning from early in ontogenesis.

## EXPERIMENT 1

Experiment 1 investigates whether preschoolers can represent abstract relations predicated on abstract, rather than perceptual dimensions. Specifically, we investigate whether 4-year-olds can succeed on a rMTS task predicated upon an abstract dimension (i.e., same and different kinds). We selected 4-year-olds as the initial age of interest for the current experiment due to previous research showing that 4-year-olds succeed at representing the abstract relations same and different in some, but not all, experimental paradigms (Hochmann et al., 2017; Kroupin & Carey, 2022b).

### Methods

#### Transparency and Openness.

All data and analytic code, as well as sample study materials, are available on the Open Science Framework at https://osf.io/6ub35/. The experiments in this paper were not preregistered.

#### Participants.

We adhered to a stopping rule of 24 participants in each age group and condition, for all experiments reported in this paper. This sample size equal to, or larger than, sample sizes used in previous experiments on children’s relational match-to-sample tasks (e.g., Christie & Gentner, 2014; Hochmann et al., 2017; Kroupin & Carey, 2022b). In Experiment 1, 24 4-year-olds (*M* = 4.62 years, *SD* = .30 years, range = 4.14–4.99 years; 13 females, 11 males) participated in the study. Researchers tested three additional 4-year-olds, whose data were excluded due to multiple, consistent experimenter errors (two) or external interference (one). All experiments reported in this paper were approved by the Committee for the Protection of Human Subjects at a U.S. university. All parents of child participants provided informed consent. All children were recruited from a university database and reflected local convenience samples (i.e., predominantly upper-middle-class and White children living in the Northeastern United States), participated in the experiment in a private laboratory room, and received a small prize for their participation. Data collection for all experiments occurred between 2015–2017.

#### Stimuli and Procedure.

We used two types of arrays, printed on small laminated flashcards: same-arrays, in which the two images showed non-identical animals belonging to the same kind (e.g., a cat standing up and a cat lying down), and different-arrays, in which the two images showed non-identical animals belonging to different kinds (e.g., a monkey and a sheep). Each card was used on only one trial, and no kind appeared on more than one array.

The cards were grouped into triads of arrays. A different triad was used on each trial. In total, there were 16 trials (eight training trials and eight test trials). Within each triad, no individual image that appeared on one of the cards appeared on either of the other two. Two of the cards in each triad were designated choice cards. Choice cards always included one same-array and one different-array. The third card in each triad was designated the sample card. During both training and test, the sample cards were always either same or different arrays. Participants were asked to select the choice card that they thought went with the sample card.

#### Training Trials.

Our rMTS task used a script identical to Hochmann et al. (2017) rMTS task. The experimenter introduced the study by saying, “We’re going to play a game. I’m going to show you some cards. Some cards go together and some cards don’t go together. I’m going to ask you to help me sort them. First I’ll teach you how to play the game.”

The experimenter then held up a same-array choice card and said, “See this card?” and placed it on the table. After the child responded affirmatively, the experimenter held up a different-array choice card and said, “See this card?” and also placed it on the table. While touching both choice cards, she said: “These cards do not go together because these cards are not alike.” Then, the experimenter held up a third, sample, card, either with a same- or different-array, and placed it on the table, saying “See this card?” Pointing back at the two choice cards, the experimenter said, “Which one of these cards goes with this [the sample] card?”

If the child made a correct response, the experimenter said, “That’s right! These cards go together because these cards are more alike” and moved onto the next trial. If the child made an incorrect response, the experimenter said, “Nice try, but actually these cards don’t go together because these cards are not alike.” The experimenter then took away the incorrect card and showed the child the two cards that matched, stating, “These cards go together because these cards are alike. In this game, you have to look at the whole card to figure out which ones go together. Do you see why these cards go together?”

Each child went through a total of eight training trials, four with a same-card sample and four with a different-card sample. Within each session the all-same choice cards appeared on the left side half the time and on the right side half of the time. In addition, the correct choice was 50% of the time the right card and 50% of the time the left card, such that a participant with a side bias would respond at chance both for all-same trials and for all-different trials. The order of all-same sample or all-different sample trials was randomized, subject to the constraint that there were no more than 3 trials of a single trial type in a row.

#### Test Trials.

The procedure of the test trials was identical to the training trials, except the experimenter stopped providing feedback. The experimenter introduced this phase by saying, “Alright, now that you know how to play the game, I’m going to let you play all by yourself! That means I’m not going to tell you which cards I think go together anymore and just let you choose.” The experimenter then proceeded through the test trials without feedback. Each child received eight test trials, four with a same-sample card and four with a different-sample card, counterbalanced as were the training trials. For the last two test trials, one with a same-sample card and one with a different-sample card, the child was asked for an explanation of his or her choice (“Why do you think this card goes with this card?”), independent of the choice being correct or incorrect.

### Results and Discussion

#### Main Analyses.

There was no difference between 4-year-olds’ performance in the Training and Test trials, *t*(46) = .89, *p* = .38. This is consistent with previous research showing that preschoolers performed similarly on a rMTS involving same and different perceptual identities on the training and test trials (Hochmann et al., 2017). Consequently, we aggregated children’s data across the two experimental phases. We found that 4-year-olds’ performance did not differ from chance levels, *M* = 51.82%, *SE* = 3.85%, *t*(23) = .47, *p* = .64. Consequently, 4-year-olds do not succeed at a rMTS involving same and different kinds. This is consistent with previous research demonstrating that 4-year-olds also do not succeed at a rMTS involving same and different perceptual identities (Hochmann et al., 2017; Kroupin & Carey, 2022a). For children’s performance on all training trials and test trials across experiments, broken down by experimental condition and age, see Table 1.

**Table 1.** Descriptive table of children’s performance across all training and test trials, by experimental condition and age.

	**4-year-olds Baseline (Exp. 1)**	**3-year-olds Familiar Noun (Exp. 2)**	**4-year-olds Familiar Noun (Exp. 2)**	**5-year-olds Familiar Noun (Exp. 2)**	**4-year-olds Novel Noun (Exp. 3)**	**5-year-olds Novel Noun (Exp. 3)**	**4-year-olds Novel Adjective (Exp. 3)**	**5-year-olds Novel Adjective (Exp. 3)**
**Training**	*M* = 48.96%	*M* = 54.69%	*M* = 61.46%	*M* = 80.73%	*M* = 67.19%	*M* = 71.88%	*M* = 53.65%	*M* = 51.04%
	*SE* = 4.25%	*SE* = 4.11%	*SE* = 5.42%	*SE* = 4.32%	*SE* = 4.24%	*SE* = 4.00%	*SE* = 4.61%	*SE* = 4.81%
**Test**	*M* = 54.69%	*M* = 52.25%	*M* = 76.04%	*M* = 82.29%	*M* = 61.27%	*M* = 76.04%	*M* = 58.85%	*M* = 56.77%
	*SE* = 4.87%	*SE* = 3.01%	*SE* = 9.06%	*SE* = 4.63%	*SE* = 4.67%	*SE* = 4.25%	*SE* = 4.90%	*SE* = 4.58%

#### Explanations.

In exploratory analyses, we investigated 1) whether children in the present experiment provided explanations appealing to sameness and difference, 2) whether children who provided explanations appealing to sameness and difference and children who provided irrelevant explanations performed above chance levels, and 3) whether children who provided explanations appealing to sameness and difference performed more accurately on the task than children who provided irrelevant explanations. All 24 participants provided a single explanation on the penultimate (i.e., seventh) trial and the final (i.e., eighth) trial, leading to a total of 48 explanations. Explanations were coded blind to participants’ performance on the test trials. Following explanation coding schemes from previous rMTS experiments (Hochmann et al., 2017), explanations were sorted into two categories: Same/Different explanations and Irrelevant explanations. Same/Different explanations appealed to the relations of sameness and difference among icons on each card, typically by mentioning the words “same” and “different” (e.g., “Because they’re the same”), kind membership (e.g., “Because there’s two birds”), or both (e.g., “Because they both have the same animals”). In contrast, Irrelevant explanations appealed to perceptual properties (e.g., “Because that one has stripes”), restated the instructions (e.g., “Because they’re more alike”), or constituted non-responses or random guessing (e.g., “I don’t know”). Two coders independently coded all 48 explanations, and intercoder reliability was 96%, converging on the same explanation category for 46 out of 48 explanations. The categorization of the remaining 2 explanations was resolved through discussion.

We found that 23 out of 24 children (96%) provided the same kind of explanation on both trials (i.e., providing either two Same/Different explanations or two Irrelevant explanations). Consequently, we categorized every participant as either a Same/Different explainer or an Irrelevant explainer. The one child who provided one Same/Different explanations and one Irrelevant explanation was categorized as a Same/Different explainer. Consequently, 8 out of 24 children (33%) were categorized as Same/Different explainers and 16 out of 24 children (66%) were categorized as Irrelevant explainers.

We found that Same/Different explainers performed at chance levels, *M* = 63.28%, *SE* = 7.87%, *t*(7) = 1.69, *p* = .14. Similarly, Irrelevant explainers also performed at chance levels, *M* = 46.09%, *SE* = 3.60%, *t*(15) = 1.08, *p* = .30. Same/Different explainers performed significantly more accurately than Irrelevant explainers, *t*(22) = 2.29, *p* = .03, but this difference was not significant after correcting for multiple comparisons (Benjamini & Hochberg, 1995). Overall, the majority of 4-year-olds provided irrelevant explanations to justify their responses. Moreover, there is no difference between Same/Different explainers and Irrelevant explainers: both kinds of explainers perform at chance levels.

## EXPERIMENT 2

Experiment 1 demonstrated that 4-year-olds do *not* succeed on a rMTS task predicated upon abstract dimensions (i.e., same and different kinds). However, previous research suggests that human adults and children may sometimes fail on rMTS tasks not due to an inability to represent the abstract relations same and different, but rather due to differences in inductive biases (Kroupin & Carey, 2021, 2022a, 2022b). In other words, participants may possess the abstract relations same and different, but fail to realize that they need to apply those relations to the experimental task at hand.

Given previous evidence of inductive challenges hindering preschoolers’ rMTS performance, Experiment 2 investigated whether noun labels might facilitate preschoolers’ performance on a rMTS predicated upon same and different kinds. Previous research suggests that language might generally facilitate relational reasoning (Christie & Gentner, 2014; Gentner, 2016; though see Hoyos et al., 2016). Moreover, given that the present experimental paradigm is a rMTS predicated upon same and different kinds, and that noun labels highlight kind membership (Dewar & Xu, 2007, 2009), noun labels might consequently highlight a relevant and helpful dimension in the present task. Overall, Experiment 2 investigated whether preschoolers might succeed in representing abstract relations predicated upon kind membership in a rMTS task, in a faciliatory condition with noun labels.

Moreover, Experiment 2 explores the developmental trajectory of preschoolers’ performance on a rMTS predicated upon same and different kinds, specifically by expanding upon the age range of the participants. Consequently, in Experiment 2, we collected data not only 4-year-olds, but also 3-year-olds and 5-year-olds.

### Methods

#### Participants.

24 3-year-olds (*M* = 3.48 years, *SD* = .31 years, range = 3.02–3.97 years; 10 females, 14 males), 24 4-year-olds (*M* = 4.48 years, *SD* = .25 years, range = 4.02–4.86 years; 8 females, 16 males), and 24 5-year-olds (*M* = 5.39 years, *SD* = .30 years, range = 5.01–5.93 years; 14 females, 10 males) participated in the study. All 4- and 5-year-olds completed the entire study. There was missing data from two of the 3-year-old participants, due to fussiness (i.e., one 3-year-old refused to complete the final test trial), and technical issues (i.e., a video recording error on two test trials for another 3-year-old), resulting in 3 missing trials out of a total of 1,152 trials.

#### Stimuli and Procedure.

Experiment 2’s stimuli and procedure were identical to Experiment 1’s stimuli and procedure, except that the experimenter also used count nouns to label each image on the eight training trials.

#### Training Trials.

Specifically, during the training trials, the experimenter would place a same-array choice card on the table and label each image while pointing to it (e.g., “See this card? That’s a cat and that’s a cat!”). Then, the experimenter would place a different-array choice card on the table and label each image while pointing to it (e.g., “See this card? That’s a sheep and that’s a monkey!”). While touching both choice cards, she said: “These cards do not go together because these cards are not alike.” Then, the experimenter would place a sample card on the table and label each image while pointing to it (e.g., “See this card? That’s an eagle and that’s a lizard!”). Pointing back at the two choice cards, the experimenter said, “Which one of these cards goes with this [the sample] card?”

If the child made a correct response, the experimenter provided positive feedback using the count noun labels (e.g., “That’s right! The card with the sheep and the monkey goes with the card with the eagle and the lizard, because these cards are alike!”). If the child made an incorrect response, the experimenter provided corrective feedback using the count noun labels (e.g., “Actually, the card with the cat and the cat doesn’t go with the card with the eagle and the lizard, because these cards are not alike. The card with the sheep and the monkey goes with the card with the eagle and the lizard, because these cards are alike. In this game, you have to look at the whole card to figure out which ones go together. Do you see why these cards go together?”).

#### Test Trials.

Experiment 2’s test trials were identical to Experiment 1’s test trials (i.e., the experimenter did not label the images were labeled or provide feedback).

### Results and Discussion

#### Main Analyses.

A repeated-measure ANOVA with Age (3 years, 4 years, 5 years) as a between-subject variable and Experimental Phase (Training, Test) as a within-subject variable revealed a main effect of Age, *F*(1, 140) = 26.47, *p* < .001. There was no effect of Experimental Phase, *F*(1, 140) = 1.05. *p* = .31, and no interaction between Age and Experimental Phase, *F*(1, 140) = .13. *p* = .71.

Since there was a main effect of Age, we then investigated each age groups’ performance against chance levels. As in Experiment 1, there was no difference in performance between training and test trials in Experiment 2, so we aggregated children’s data across the two experimental phases. We found that 3-year-olds’ performance did not differ from chance, *M* = 53.47%, *SE* = 2.60%, *t*(23) = 1.33, *p* = .20. However, 4-year-olds performed at better than chance levels, *M* = 68.75%, *SE* = 5.67%, *t*(23) = 3.31, *p* = .003. Similarly, 5-year-olds performed at better than chance levels, *M* = 81.51%, *SE* = 3.68%, *t*(23) = 8.57, *p* < .001. Moreover, we also compared 4-year-olds’ performance between Experiment 1 (i.e., the baseline condition) and Experiment 2 (i.e., the noun label training condition). 4-year-olds provided with noun label training in Experiment 2 performed significantly more accurately than the 4-year-olds in the baseline condition in Experiment 1, *t*(46) = 2.47, *p* = .02. All significant results remained significant after correcting for multiple comparisons (Benjamini & Hochberg, 1995).

Thus, while Experiment 1 showed that 4-year-olds do not succeed at a rMTS involving same and different kinds at baseline, Experiment 2 showed that a brief training phase involving noun labels led to an improvement in 4-year-olds’ performance at a rMTS involving same and different kinds. Consequently, noun labels may play a faciliatory role, at least in the present task. Moreover, Experiment 2 articulates the developmental trajectory of preschoolers’ capacity to represent abstract relations predicated on same and different kinds, demonstrating that 3-year-olds fail a rMTS task with same and different kinds with faciliatory noun label training, whereas 4- and 5-year-olds succeed in the task.

#### Explanations.

We once again examined participants’ explanations, specifically investigating 1) whether children in the present experiment provided explanations appealing to sameness and difference, 2) whether children who provided explanations appealing to sameness and difference and children who provided irrelevant explanations performed above chance levels, and 3) whether children who provided explanations appealing to sameness and difference performed more accurately on the task than children who provided irrelevant explanations. All 72 participants provided a single explanation on the penultimate (i.e., seventh) trial and the final (i.e., eighth) trial, except for one 3-year-old who fussed out on the final trial, leading to a total of 143 explanations. Explanations were coded blind to participants’ performance on the test trials, and explanations were once sorted into two categories: Same/Different explanations (e.g., “Because they’re the same”; “because these two are different and these two are different”; “Because they’re not the same bugs, because they’re different”; “Because there’s a flamingo and a penguin and a dolphin and a bear”) and Irrelevant explanations (e.g., “Because this one is basically doing what this one is doing”; “Because I do”; “I don’t know”). Children in all age groups provided both Same/Different and Irrelevant explanations.

Two coders independently coded all 143 explanations, and intercoder reliability was 97%, converging on the same explanation category for 138 out of 143 explanations. The categorization of the remaining 2 explanations was resolved through discussion. 56 out of 72 children (78%) provided the same kind of explanation on both trials (i.e., providing either two Same/Different explanations or two Irrelevant explanations). 15 out of 72 children (21%) provided one Same/Different explanations and one Irrelevant explanation, and were also categorized as Same/Different explainers. The one 3-year-old who fussed out in the last trial provided an Irrelevant explanation on the penultimate trial, and as categorized as an Irrelevant explainer. Consequently, 49 out of 72 children (68%) were categorized as Same/Different explainers and 23 out of 72 children (32%) were categorized as Irrelevant explainers.

In the entire sample of 3- to 5-year-olds, we found that Same/Different explainers performed at significantly above chance levels, *M* = 72.87%, *SE* = 3.11%, *t*(48) = 7.35, *p* < .001. In contrast, Irrelevant explainers performed at chance levels, *M* = 57.34%, *SE* = 4.84%, *t*(22) = 1.52, *p* = .14. Moreover, Same/Different explainers performed significantly more accurately than Irrelevant explainers, *t*(70) = 2.76, *p* = .007. All significant statistics remained significant after correcting for multiple comparisons (Benjamini & Hochberg, 1995). Overall, exploratory analyses of children’s explanations showed that children frequently provided explanations appealing to sameness and difference to justify their responses, and that children who appealed to sameness and difference performed more accurately on the experimental task.

## EXPERIMENT 3

Experiment 1 showed that 4-year-olds fail on a rMTS predicated upon same and different kinds without noun labels. However, Experiment 2 showed that 4- and 5-year-olds, but not 3-year-olds, succeed on the same rMTS predicated upon same and different kinds when given training with noun labels. Consequently, noun labels may be beneficial for preschoolers’ relational reasoning capacities, in experimental paradigms where the noun labels highlight the relevant dimension (i.e., kind membership) that form the basis of same and different relations.

While noun labels might facilitate preschoolers’ performance on a rMTS predicated upon same and different kinds by highlighting kind membership, a deflationary alternative is that simple verbal repetition (e.g., hearing “lion” and “lion” while looking at pictures of two lions) facilitated preschoolers’ performance by highlighting the sameness of the objects. Thus, an outstanding question is whether noun labels’ psychological properties uniquely facilitate preschoolers’ relational reasoning, or if facilitation could occur with any sets of repeated words.

Consequently, Experiment 3 adjudicates between these two possibilities by contrasting children’s performance on rMTS task with noun labels or adjective labels. Children participated in a rMTS with novel kinds (i.e., later-generation Pokemon that are generally unfamiliar and unnamable to a general audience). The experimenter will provide either novel noun labels (e.g., “This one is a dax and this one is a dax!”) or novel adjectives (e.g., “This is a daxy one and this is a daxy one!”). If count noun labels uniquely highlight category membership, the relevant dimension of sameness and difference in this relational reasoning task, then children might succeed in the novel noun condition but fail in novel adjective condition. In contrast, if the deflationary alternative—specifically, that any form of verbal repetition will sufficiently highlight sameness—is correct, then children should succeed equally in both the novel noun and adjective conditions.

Since most adjectives frequently used to describe familiar animals (e.g., “fluffy”, “cute”, “small”) are also typically true of multiple kinds of animals (e.g., cats, dogs, hamsters, and rabbits can all be fluffy, cute, and small), we used novel adjectives (e.g., “daxy”, “feppish”) in the current experimental paradigm. Consequently, we also used novel nouns that closely matched the novel adjectives (e.g., “dax”, “fep”). Since children possess a mutual exclusivity bias (Halberda, 2003; Markman et al., 2003) and may consequently reject novel noun labels for familiar kinds that they have already mapped labels onto, we used novel kinds (i.e., later-generation Pokemon). Moreover, since Experiment 2 shows that 4- and 5-year-olds, but not 3-year-olds, pass a rMTS predicated upon same and different kinds in a facilitatory noun label condition, Experiment 3 focuses on only 4- and 5-year-olds. Overall, Experiment 3 not only investigates whether noun labels uniquely facilitate preschoolers’ relational reasoning abilities over and above simple verbal repetition, but also uses another set of stimuli to potentially provide converging evidence for Experiment 2’s result that noun labels facilitate preschoolers’ relational reasoning abilities.

### Methods

#### Participants.

48 4-year-olds (*M* = 4.46 years, *SD* = .30 years, range = 4.01–4.95 years; 36 females, 32 males), and 48 5-year-olds (*M* = 5.45 years, *SD* = .29 years, range = 5.01–5.99 years; 23 females, 25 males) participated in the study. We removed trials in which children spontaneously recognized the superordinate category (i.e., Pokemon) from the analyses, because this recognition indicated that the stimuli were less novel to these children than to other children. A few children spontaneously recognized the superordinate category across all training and test trials, and their data were excluded altogether. Consequently, researchers tested four additional 4-year-olds, whose data were excluded due to multiple, consistent experimenter errors (one), fussiness (one) or spontaneously recognizing the superordinate category (two), as well as five additional 5-year-olds, whose data were excluded because they spontaneously recognized the superordinate category (i.e., Pokemon) across all trials. Moreover, there was missing data from five of the 4-year-old participants, due to experimenter error (i.e., experimenter error on a single test trial for two 4-year-olds), superordinate category recognition (i.e., one 4-year-old on a single training trial, one 4-year-old on a single test trial, and one 4-year-old on a single training trial and a single test trial), as well as missing data from two of the 5-year-old participants due to superordinate category recognition (i.e., one 5-year-old who recognized the superordinate category on one training trial, one 5-year-old who recognized the superordinate category on two test trials), resulting in 9 missing trials out of a total of 1,536 trials.

#### Stimuli and Procedure.

We used two types of arrays, printed on small laminated flashcards: same-arrays, in which the two images showed non-identical animals belonging to the same kind, and different-arrays, in which the two images showed non-identical animals belonging to different kinds. Each card was used on only one trial, and no kind appeared on more than one array. Critically, the images presented during both the training and test phases depicted novel kinds rather than known kinds.

#### Training Trials: Novel Noun Label Condition.

Experiment 3’s novel noun training trials were highly similar to Experiment 2’s training trials, except using novel rather than familiar kinds. Each novel kind was labeled with a novel noun label rather than a known noun label. In other words, the experimenter introduced same-array and different-array cards with novel noun labels (e.g., “See this card? This one’s a dax and this one’s a dax!” to introduce a same-array card; “See this card? This one’s a fep and this one’s a wug!” to introduce a different-array card). Similarly, the experimenter also provided feedback with novel noun labels (i.e., “That’s right! The card with the dax and the dax goes with the card with the blick and the blick, because these cards are alike!” in response to a correct response from the child; “Actually, the card with the fep and the wug doesn’t go with the card with the blick and the blick, because these cards are not alike. The card with the dax and the dax goes with the card with the blick and the blick, because these cards are alike. In this game, you have to look at the whole card to figure out which ones go together. Do you see why these cards go together?” in response to an incorrect response from the child).

#### Training Trials: Novel Adjective Label Condition.

Experiment 3’s novel adjective training trials were identical to Experiment 3’s novel noun training trials, except that each novel kind was labeled with a novel adjective label rather than a novel noun label (e.g., “This is a daxy one and this is a daxy one” rather than “This one’s a dax and this one’s a dax”; “This is a feppish one and this is a wuggy one” rather than “This one’s a fep and this one’s a wug”).

#### Test Trials.

Experiment 3’s test trials were identical to Experiment 2’s test trials, except the images on the cards depicted novel kinds rather than known kinds.

### Results and Discussion

#### Main Analyses.

A repeated-measure ANOVA with Age (4 years, 5 years) and Condition (Noun, Adjective) as between-subject variables, and Experimental Phase (Training, Test) as a within-subject variable revealed a main effect of Condition, *F*(1, 184) = 19.25, *p* < .001. There was no main effect of Age, *F*(1, 184) = 1.34, *p* = .25, or of Experimental Phase, *F*(1, 184) = .52, *p* = .47. There was a marginal interaction between Age and Condition, *F*(1, 184) = 3.57, *p* = .06. All other interactions were not significant (all *p*’s > .32).

**Figure 1.**
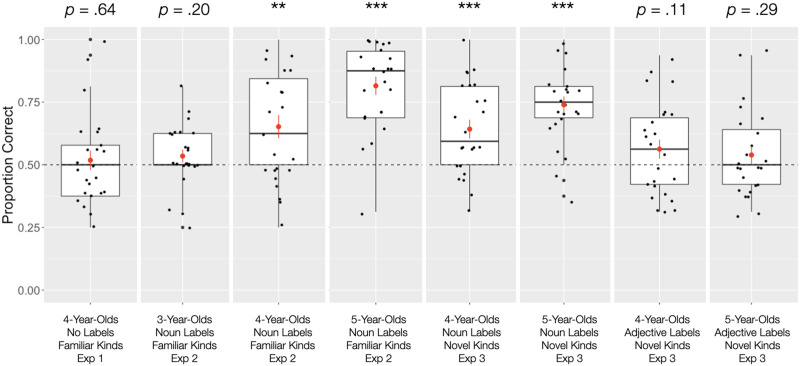
Proportion of correct responses by age group and condition, across all three experiments. The vertical length of each box indicates the interquartile range, and the horizontal line represents the median. The red dot is the mean. Error bars show 1 *SD* and whiskers show the entire range of responses. Dots indicate individual data.

Since there was a main effect of Condition, we then investigated each condition against chance levels. As in Experiments 1 and 2, there was no difference in performance between training and test trials in Experiment 3, so we aggregated children’s data across the two experimental phases. We found that 4- and 5-year-olds in the Novel Noun Label condition performed at better than chance levels, *M* = 69.10%, *SE* = 2.58%, *t*(47) = 7.41, *p* < .001. In contrast, 4- and 5-year-olds in the Novel Adjective Label condition performed at chance levels, *M* = 55.08%, *SE* = 2.59%, *t*(47) = 1.96, *p* = .06. All significant results remained significant after correcting for multiple comparisons (Benjamini & Hochberg, 1995). For a visualization of data across all three experiments, see Figure 1.

Thus, Experiment 3 showed that the facilitatory effect of labeling is specific to noun labels, by demonstrating that noun labels, but not adjective labels, facilitate preschoolers’ performance on a rMTS involving same and different kind relations. Consequently, the faciliatory effect of noun labels cannot be explained by a lower-level mechanism that applies to many kinds of words or sounds (i.e., a simple repetitive phenomena). Rather, the faciliatory effect of noun labels may be due to the fact that noun labels encourage preschoolers to attend to kind membership (Gelman, 2003), the relevant dimension upon which the task is predicated.

#### Explanations.

We once again examined participants’ explanations by investigating 1) whether children in the present experiment provided explanations appealing to sameness and difference, 2) whether children who provided explanations appealing to sameness and difference and children who provided irrelevant explanations performed above chance levels, and 3) whether children who provided explanations appealing to sameness and difference performed more accurately on the task than children who provided irrelevant explanations. 94 participants provided a single explanation on both the penultimate (i.e., seventh) trial and the final (i.e., eighth) trial, and the experimenter forgot to ask for explanations from two children (i.e., one 4-year-old and one 5-year-old in the novel adjective conditions) leading to a total of 188 explanations. Explanations were coded blind to participants’ performance on the test trials, and explanations were once sorted into two categories: Same/Different explanations (e.g., “Because they look more like the same”; “They both have two different animals”; “Because they have different things”) and Irrelevant explanations (e.g., “Because they both have tips”, “Because these are both lovely and not scary”; “Because they’re both closing their eyes”; “Because they’re friends”; “I don’t know”). Unlike Experiments 1 and 2, children in Experiment 3 did not provide explanations appealing to kind membership because they did not know the names of the animals. Children in both age groups and conditions provided both Same/Different and Irrelevant explanations.

Two coders independently coded all 188 explanations, and intercoder reliability was 93%, converging on the same explanation category for 176 out of 188 explanations. The categorization of the remaining 12 explanations was resolved through discussion. 83 out of 94 children (88%) provided the same kind of explanation on both trials (i.e., providing either two Same/Different explanations or two Irrelevant explanations). 11 out of 94 children (12%) provided one Same/Different explanations and one Irrelevant explanation, and were also categorized as Same/Different explainers. Consequently, 45 out of 94 children (48%) were categorized as Same/Different explainers and 49 out of 94 children (52%) were categorized as Irrelevant explainers.

In the entire sample of 4- and 5-year-olds across both conditions, we found that Same/Different explainers performed at significantly above chance levels, *M* = 72.64%, *SE* = 2.63%, *t*(44) = 8.61, *p* < .001. In contrast, Irrelevant explainers performed at chance levels, *M* = 52.89%, *SE* = 2.22%, *t*(48) = 1.30, *p* = .20. Moreover, Same/Different explainers performed significantly more accurately than Irrelevant explainers, *t*(92) = 5.77, *p* < .001. All significant statistics remained significant after correcting for multiple comparisons (Benjamini & Hochberg, 1995).

Consequently, exploratory analyses of children’s explanations in Experiment 3 show a similar pattern to exploratory analyses of children’s explanations in Experiments 1 and 2. Specifically, Same/Different explainers perform above chance levels, Irrelevant explainers perform at chance levels, and Same/Different explainers perform significantly more accurately on Irrelevant explainers.

## GENERAL DISCUSSION

The present research builds on upon a large literature demonstrating children’s early-emerging relational reasoning abilities (Anderson et al., 2018; Christie & Gentner, 2014; Goddu et al., 2020; Hochmann et al., 2016, 2017; Walker et al., 2016; Walker & Gopnik, 2017), by showing that preschoolers can represent not only relations of sameness and difference predicated on perceptual dimensions (i.e., size, shape, color), but also relations of sameness and difference predicated on abstract dimensions (i.e., kind membership). While Experiment 1 showed that 4-year-olds fail on a rMTS involving familiar kinds, Experiment 2 showed that 4- and 5-year-olds, but not 3-year-olds, succeed on the same rMTS involving familiar kinds when provided with noun label training. Furthermore, Experiment 3 showed that 4- and 5-year-olds also succeed on a rMTS involving novel kinds when provided with noun label training, but not when provided with adjective label training. Taken together, these experiments demonstrate that preschoolers possess sophisticated relational reasoning capacities. Moreover, noun labels might uniquely facilitate young children’s relational reasoning capacities, at least in some contexts.

Moreover, we found that preschoolers provided informative explanations when asked to justify their responses, on both the rMTS involving familiar kinds and the rMTS involving novel kinds. We categorized each participant as appealing to sameness and difference in their explanations (e.g., “Because they’re not the same bugs, because they’re different”; “Because these two are different and these two are different”; “Because there’s two of the same thing”), or as appealing to irrelevant information in their explanations (e.g., “Because they’re all standing up”; “Because it’s actually a robot”; “I forget”; “I don’t know”). In Experiments 2 and 3, children who appealed to sameness and difference in their explanations performed significantly above chance levels on the rMTS, whereas children who appealed to irrelevant explanations performed at chance levels. Moreover, in Experiments 2 and 3, children who appealed to sameness and difference in their explanations performed significantly more accurately than children who appealed to irrelevant explanations. These findings are consistent with previous research showing that preschoolers who succeeded on a rMTS predicated upon sameness and difference of perceptual identity more frequently justified their responses by appealing to the words “same” and “different”, relative to preschoolers who failed at the same rMTS predicated upon sameness and difference (Hochmann et al., 2017), as well as a larger body of literature showing that preschoolers who succeed on experimental tasks can often explicitly justify their responses (e.g., Zhu & Gopnik, 2023, 2024). It is unclear whether possessing the capacity to verbally state a rule, or to understand the words “same” and “different”, are necessary conditions for succeeding on a rMTS task; future research may explore the relation between children’s capacity to understand the words “same” and “different” and their capacity for relational reasoning more closely. In the current experiments, the nature of children’s explanations provided additional evidence suggesting that children succeeded on a rMTS predicated upon same and different kind membership by leveraging their representations of sameness and difference.

Moreover, we found that training with noun labels facilitated 4- and 5-year-olds’ success on rMTS. Specifically, Experiment 2 found that training with noun labels facilitated 4- and 5-year-olds’ success on rMTS involving familiar kinds. Building upon these results, Experiment 3 further found that training with noun labels, but not adjective labels, facilitated 4- and 5-year-olds’ success on rMTS involving novel kinds. These results suggest that simple verbal repetition (i.e., hearing the same word repeated while looking at two pictures belonging to the same kind) did not help preschoolers notice the sameness and difference of the items within the rMTS, Indeed, adjective labels frequently point to perceptual dimensions, which might distract children from noticing the task’s relevant dimension (i.e., kind membership). These findings support previous research suggesting that 4-year-olds’ rMTS failures may be due to differences in inductive biases rather than absence of the capacity to represent abstract relations (Kroupin & Carey, 2021, 2022b). In the present context, it is possible that noun labels facilitated children’s success on a relational reasoning task, potentially by changing their inductive biases. Thus, the present experiments provide further support for the general claim that language can facilitate relational reasoning (Christie & Gentner, 2014; Gentner, 2016).

Might 4- and 5-year-olds succeed on a rMTS involving kind membership not through the representation of abstract relations (i.e., sameness and difference) along abstract dimensions (i.e., kind membership), but through lower-level perceptual strategies? In previous rMTS involving 16 items of same or different perceptual identities, young children and some animals (i.e., baboons, pigeons) succeeded on the experimental task not through abstract rules such as “all same” or “all different”, but through perceptual cues like entropy (Fagot et al., 2001; Hochmann et al., 2017; Wasserman et al., 2000, 2001; Wasserman & Young, 2010; Young et al., 1997). Consequently, we must consider whether preschoolers in the present task might leverage lower-level perceptual cues (i.e., perceptual similarities or dissimilarities between objects in pairs) to succeed on a rMTS involving kind membership. Indeed, two members belonging to the same kind are typically more perceptually similar than two members belonging to different kinds (e.g., Diesendruck & Bloom, 2003). However, we believe that preschoolers in the present tasks are unlikely to succeed using on a lower-level strategy based on perceptual similarity, given previous research demonstrates that 4-year-olds typically fail rMTS involving perceptually identical items (Hochmann et al., 2017). Given this previous research showing that 4-year-olds fail at a rMTS involving perceptually identical items and perceptually non-identical items, it is not surprising that 4-year-olds also fail at a rMTS involving perceptually similar items (i.e., belonging to the same kind) and perceptually dissimilar items (i.e., belonging to different kinds). Moreover, Experiment 3’s finding that noun labels, but not adjective labels, facilitate 4- and 5-year-olds’ performance on a rMTS predicated upon kind membership tentatively suggests that preschoolers are attending to the abstract conceptual information highlighted by noun labels, rather than lower-level perceptual information highlighted by any kind of simple verbal repetition. Additionally, in the noun label conditions, preschoolers often appealed to kind membership in their explanations, both when presented with familiar kinds (e.g., “Because these are both donkeys and these are both lobsters”; “Because the spider and the snail goes with the owl and the alligator”; “Because they’re not the same animals”) and when presented with novel kinds (e.g., “Because I think they’re the same animal”; “Because they both have two different animals”), suggesting that 4- and 5-year-olds possess a genuine capacity to represent sameness and difference of kind membership. However, future research might further rule out the lower-level perceptual account by using a rMTS involving items that belong to the same kind, but that are also perceptually dissimilar (e.g., an apple and a banana; Gentner & Namy, 1999). Further empirical work demonstrating that preschoolers succeed on a rMTS predicated on same and different kinds, even in the absence of lower-level visual similarity cues, would bolster the present claim that preschoolers can reason about abstract relations based on abstract dimensions.

While the present experiments demonstrate that, with noun label training, 4- and 5-year-olds can represent the abstract relations same and different predicated upon the abstract dimension of kind membership, there are still many open questions for future research. For example, it is unclear *why* 3-year-olds failed on the rMTS predicated on kind membership even with noun label training, while 4-year-olds succeeded on the same task. There are multiple possibilities regarding what cognitive abilities might be developing between the ages of three and four years, and these possibilities are not mutually exclusive. One possibility is that relational reasoning abilities are still developing between three and four years of age. For example, 4-year-olds, but not 3-year-olds, succeed at a standard rMTS predicated upon perceptual similarities and differences (Hochmann et al., 2017). Consequently, it is unsurprising that 4-year-olds, but not 3-year-olds, also succeed on the present task, a rMTS predicated upon abstract similarities and differences. Another possibility is that a full-fledged understanding of kind representations is still developing between three and four years of age. While even infants possess some understanding of the privileged relation between noun labels and kind membership (Dewar & Xu, 2007, 2009), particular aspects of a theory of kinds, such as a sensitivity to animals’ insides and essences, develops around four years but not earlier (Gelman, 2004; Gelman & Wellman, 1991). Thus, 3-year-olds’ developing theories of natural kinds may not be sufficiently developed to support successful performance on a rMTS predicated upon same and different kinds. Moreover, noun labels facilitated 4- and 5-year-olds’ task performance, but did not facilitate 3-year-olds’ task performance. We suggest that noun labels facilitate participants’ performance by shifting their inductive biases, rather than by create new cognitive capacities. Consequently, noun label training may help 4- and 5-year-olds, who already possess the capacity to represent abstract relations predicated on abstract dimensions, attend to the relevant features of the present rMTS task. In contrast, if 3-year-olds do not yet possess the capacity to represent abstract relations predicated on abstract dimensions—either due to difficulties in their relational reasoning or their understanding of kind representations—then noun labels will not facilitate 3-year-olds’ performance on the present rMTS task. Overall, more theoretical and empirical work is required to articulate exactly which aspects of a theory of kinds 3-year-olds are missing, and how this might affect their subsequent rMTS performance.

Moreover, in order to maximize children’s opportunities for success on a rMTS involving same and different kinds, future iterations of this experimental paradigm might consider providing children with explicit instructions of how to sort the arrays (i.e., by telling children that “cards with the same animals go together” and “cards with different animals go together”). Surprisingly few relational reasoning paradigms explicitly provide sorting rules involving the words “same” and “different” to children (Carstensen et al., 2019; Goddu et al., 2020; Hochmann et al., 2017; Walker et al., 2016; Walker & Gopnik, 2017; though see Christie & Gentner, 2014), perhaps because researchers frequently use relational reasoning paradigms that are applicable to both humans and non-human animals (e.g., Hochmann et al., 2017), or because it is unclear when children first begin to understand the words “same” and “different” (Christie & Gentner, 2014; Hochmann et al., under review). Indeed, Hochmann et al. (under review) find that most English-speaking 4-year-olds, but only half of English-speaking 3-year-olds, understand the words “same” and “different”. Consequently, a rMTS paradigm that relies on explicit instructions may be a better paradigm to measure relational reasoning capacities in older preschoolers who already understand the words “same” and “different”, but may also introduce a negative bias against 3-year-olds who do not yet understand the words “same” and “different.” While experimental paradigms that provide children with direct, explicit instructions involving the words “same” and “different” rely on children actually understanding the words “same” and “different”, these direct, explicit paradigms may be another interesting method of assessing young children’s capacity to represent abstract relations predicated on abstract dimensions.

Additionally, the current set of experiments tested only 4-year-olds, but not 3- or 5-year-olds, in the familiar kinds baseline condition without noun label training (Experiment 1). Future research could investigate preschoolers’ performance on a kind rMTS without noun label training across a greater age range, to better understand the developmental trajectory of children’s relational reasoning abilities. Furthermore, future research with more diverse populations is required to determine the replicability and generalizability of our findings (Cao et al., 2024). There is evidence of cross-cultural variation in the development of relational reasoning (Carstensen et al., 2019), and children growing up in other cultures and contexts might demonstrate differing developmental trajectories in the acquisition of relational reasoning capacities. A limitation of our current work is its reliance on WEIRD (i.e., Western, educated, industrialized, rich, and Democratic; Henrich et al., 2010) convenience samples. Future work should explore not only the possibility of early variation in children’s abilities to reason about relations along abstract dimensions, but also potential specific cultural and contextual factors that might cause this variation.

In summary, the present series of experiments contributes to a growing body of literature demonstrating an early-emerging capacity for abstract relational reasoning in infants, toddlers, and preschoolers (Anderson et al., 2018; Brockbank et al., 2023; Carstensen et al., 2019; Christie & Gentner, 2014; Goddu et al., 2020; Hochmann, 2022; Hochmann et al., 2016, 2017; Holyoak et al., 1984; Kroupin & Carey, 2022b; Walker et al., 2016; Walker & Gopnik, 2017). The present research builds on prior research by demonstrating that, when provided with noun label training, 4- and 5-year-olds can represent not only abstract relations predicated upon perceptual dimensions, but also abstract relations predicated upon abstract dimensions, such as kind membership. Thus, relational reasoning may be a remarkably powerful and flexible learning mechanism from early childhood onwards.

## ACKNOWLEDGMENTS

I am deeply grateful to Susan Carey, a brilliant, wise, and generous mentor who encouraged me to pursue my own independent research project when I was a lab manager, and then again encouraged me to write up the research as a solo author years later. I am also grateful to Megan Dempster for assistance with data collection and Lily Zihui Zhu for assistance with explanation coding. Thanks also to the parents and children who made this research possible.

## DATA AVAILABILITY STATEMENT

The anonymized data and R code for all experiments are made public through Open Science Framework (https://osf.io/6ub35/).
